# Perceptual consensus on cross-country ski–snow performance: a questionnaire study of experts and non-experts

**DOI:** 10.3389/fspor.2026.1766019

**Published:** 2026-02-10

**Authors:** Anton Kalén, Joakim Abrahamsson, Kalle Kalliorinne, Hans-Christer Holmberg, Andreas Almqvist

**Affiliations:** 1 Digital Services and Systems, Department of Computer Science, Electrical and Space Engineering, Luleå University of Technology, Luleå, Sweden; 2 Swedish Olympic Committee, Stockholm, Sweden; 3 Student, Researcher and Educational Support, Professional Services, Luleå University of Technology, Luleå, Sweden; 4 Machine Elements, Department of Engineering Sciences and Mathematics, Luleå University of Technology, Luleå, Sweden; 5 The Swedish Research Centre for Sports and Performance Technology (SPORTC), Luleå University of Technology, Luleå, Sweden

**Keywords:** agreement analysis, expertise, glide performance, perception, ski preparation, skiing, snow, tribology

## Abstract

**Introduction:**

Understanding how skiers and ski technicians perceive snow conditions and ski preparation is essential for optimising glide performance in cross-country (XC) skiing. While tribological research has established how snow microstructure, water-film formation, and ski-snow interactions influence friction, comparatively little is known about how skiers and ski technicians interpret these mechanisms in practice.

**Methods:**

We therefore conducted a nationwide questionnaire study (*n* = 249) to quantify perceptual consensus on ten topics related to snow type, glide determinants, ski preparation, and skier-equipment interaction. Responses were analysed using van der Eijk's agreement coefficient for ordinal data and Cliff's delta to evaluate expert (*n* = 20) versus non-expert (*n* = 229) differences.

**Results:**

Agreement varied systematically across topics: the highest consensus was found for perceived ski speed on different snow types and structure selection, while the lowest was observed for glide determinants, paired glide tests involving skier mass, and double-poling positioning. Experts showed higher within-group agreement for perceived ski speed in skate-skiing and on moist transformed snow, and they consistently rated ski camber as more important than non-experts did.

**Discussion:**

These findings highlight empirically developed know-how within the skiing community, such as shared heuristics for snow-type assessment and preparation, and systematic expertise-related differences in prioritising fundamental ski characteristics (e.g., camber). As such, they can be used for targeted education and to inform future applied tribology research.

## 1 Introduction

At the systems level, cross-country (XC) skiing performance reflects the balance between propulsive and resistive forces. Under typical race conditions, ski–snow friction and aerodynamic drag account for approximately 30% and 15%, respectively, of the energetic cost [1]. The relative importance of these losses varies with the type of skiing, i.e., classic or skate, sub-technique execution, weather conditions, terrain segment, and speed [2–6]. Ski-snow friction tends to dominate resistive forces at lower speeds and during uphill work, whereas aerodynamic drag becomes increasingly important on flats and downhills as speed increases. Furthermore, the relative contribution of these forces differs between classic and skating sub-techniques due to variations in posture and speed profiles [7, 8]. Even small reductions in ski–snow friction can therefore yield meaningful performance gains. For example, a power-balance simulation of XC skiing showed that reducing the friction coefficient from 0.037 to 0.030 shortened a 4.2 km race by 48 s [2, 9], corresponding to roughly 1.6 s per kilometre for each 0.001 decrease in friction. These sensitivities highlight why glide optimisation is central to skiing performance.

In practice, glide-optimisation decisions are also made in both training and competition contexts, where available time, fatigue, and feedback quality can differ substantially. Moreover, optimisation of glide is not merely a technical task, but a systemic one. Already in 1980, Ekström characterised cross-country skiing as a “relationship between man, equipment and environment” in which all factors must be tuned to one another for an optimal result [10]. In practical terms, this framing emphasises the “human factor”, i.e., the technician’s and athlete’s ability to perceive environmental cues and adapt equipment accordingly. This ability serves as a decisive filter that links physical conditions to realised performance [11, 12].

Much of ski preparation is informed by subjective glide testing. Such testing is central but inherently sensitive to factors such as fatigue, motivation, pacing, and rapidly changing micro-conditions, which can confound perceived differences. At the same time, advances in measurement technology, e.g., differential GNSS/IMU approaches for on-snow performance analysis [13, 14] and tribometer-based quantification of ski–snow friction [3, 4, 15, 16], create opportunities to complement subjective assessment with more objective feedback and decision support under variable conditions.

The physical mechanisms governing glide and grip have been extensively studied within tribology, where ski–snow friction is known to depend on snow microstructure, water film formation, snow deformation, ski-base topography, and camber mechanics [17–28]. Recent advances have clarified how snow type, grain size, temperature, and contamination interact with ski structure, wax, and hand structuring to influence frictional behaviour [11, 19, 22, 29–32]. These insights underpin modern ski preparation practices and influence equipment choices across all performance levels [11, 30].

Despite the technical foundation, ski preparation in practice relies not only on physical principles but also on empirical knowledge developed by skiers and ski technicians [11, 30]. Skiers and ski technicians routinely make judgements about structure choice, wax types^1^ and hardness, the relative skiing speed on different snow types, and how factors such as body mass or positioning affect glide [23, 24, 26, 31]. Such decisions play a decisive role in both training and competitive environments, yet empirical evidence on how the skiing community perceives these factors is sparse [11].

Earlier work in sports science has shown that practitioners often apply heuristic reasoning under uncertainty [33, 34], and that expert decision-making can differ systematically from that of recreational participants [35]. In skiing, technical expertise in ski preparation is typically acquired through repeated exposure to diverse snow conditions and detailed feedback during waxing and testing [11, 36]. Despite this, the degree of consensus regarding key determinants of ski performance remains largely unquantified. It is unclear whether consensus exists regarding perceived ski speed on common snow types, how skiers perceive the influence of weight or positioning on glide, or how they prioritise different ski properties when aiming to optimise performance.

Ski preparation is further challenged by snow variability, rapidly changing temperature and humidity, and limited testing time. For example, even well-resourced teams encountered significant difficulties under atypical warm/wet conditions during the Sochi 2014 Winter Olympics. Understanding these perceptions is relevant for three reasons:

Agreement reflects shared mental models within the skiing community. High consensus suggests that practitioners converge on similar interpretations of snow behaviour, whereas low consensus indicates divergent conceptualisations that may influence preparation strategies [35, 36].
Quantifying perception differences between experts and non-experts provides insight into how experience shapes understanding of ski–snow interaction. Such knowledge may inform technician training, support coach–technician communication, and improve knowledge transfer between elite and recreational environments [33, 35].
Identifying areas of low agreement can expose domains where intuition deviates from physical evidence, guiding targeted educational interventions and highlighting priorities for future experimental research [11, 34].

To address these gaps, we conducted a nationwide questionnaire study to examine how Swedish skiers and ski technicians evaluate snow types, the determinants of glide performance, and ski preparation strategies. We quantified within-group agreement and between-group differences using statistical methods tailored for ordinal data [37–39]. Specifically, we sought to answer three research questions:

1. To what extent do skiers and ski technicians agree on key questions relevant to ski choice, waxing strategy, and skiing performance?
2. Does consensus vary across snow types?
3. Do experts and non-experts differ in their views on what determines skiing performance?

This study provides the first systematic mapping of perceptual consensus on ski–snow interaction across a broad sample of the skiing community. The findings clarify where experiential knowledge aligns with established physical mechanisms and where it diverges, offering a foundation to strengthen evidence-based preparation strategies and guide future applied tribology research.

## 2 Materials and methods

### 2.1 Study design and recruitment

A cross-sectional online questionnaire was distributed to Swedish skiers, coaches, and ski technicians at all levels between October 13, 2021, and January 31, 2022. The questionnaire link was disseminated through local ski clubs, regional skiing networks, and public sport-related social media channels. Participation was voluntary and anonymous, and no incentives were offered. Respondents were eligible if they were active skiers (classic and/or skate) and at least 15 years of age. No additional exclusion criteria were applied. In accordance with the Swedish Ethical Review Act, this study did not require ethical approval as it involved only questionnaire data collection with no processing of personal information, no physical intervention, and no methods designed to physically or psychologically affect the participants. Participants provided informed consent via the survey platform and could withdraw at any time prior to submitting the questionnaire by closing the survey window.

A total of 249 participants completed the questionnaire. We categorised participants as experts (*n* = 20, 8%) if they met the following criteria:

Over 500 ski preparations per year.
Over 1,000 km of skiing per year.
Reported a role as waxer.

The remaining participants were classified as non-experts (*n* = 229, 92%). This classification was established *post-hoc* in consultation with national team ski technicians from XC skiing and biathlon to ensure a sufficiently high threshold was established to be considered an expert.

### 2.2 Questionnaire structure

The questionnaire addressed various aspects of ski preparation and glide perception across eight distinct snow conditions. The snow conditions were classified according to SWIX’s snow classification system (as of October 13, 2021), presented with icons in Table 1. Note that this system has been refined since the questionnaire administration. The list of questions and answer options is detailed in Table 2.

**Table 1:** The questionnaire’s snow types ST1–ST8, and the correspondence to SWIX’s nine field types as icons (adapted and recolored from the originals).

ST#	Questionnaire	Icon(s)	Note
ST1	New snow	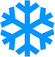 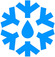	Maps to both dry and moist new snow.
ST2	Fine-grained snow	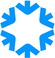	Dry, rounded fine grains.
ST3	Transformed snow	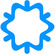	Old/rounded, dry grains.
ST4	Frozen wet snow	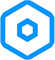	Refrozen melt–freeze grains.
ST5	Moist fine-grained snow	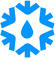	Fine grains with free water.
ST6	Moist transformed snow	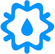	Transformed grains with free water.
ST7	Wet corn snow	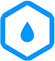	Melt–freeze grains, liquid water present.
ST8	Very wet corn snow	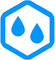	Saturated corn/slush.

**Table 2:** Translated full wording of the ten questionnaire items (Q1–Q10) with response options.

Q#	Question	Answers
Q1	In classic skiing, how fast do you perceive the conditions to be for each snow type?	Very slow, Slow, Equal, Fast, Very fast
Q2	In skate skiing, how fast do you perceive the conditions to be for each snow type?	Very slow, Slow, Equal, Fast, Very fast
Q3	Two skiers have identical skis; one weighs 70 kg and the other 85 kg. In each snow condition, who wins a paired glide test? (Assume aerodynamic drag is negligible.)	70 kg skier, Tied, 85 kg skier
Q4	Based on your own experience, which grip system works best on classic skis for each snow type?	Hard wax, Hard wax + Klister, Klister + Hard wax, Klister, Roughened base ∼ “Rugg”
Q5	When double poling, where, in each snow condition, should you position yourself on the groomed track to achieve the best glide?	In the classic tracks, It does not matter, Outside the classic tracks
Q6	You are skate skiing on a sunny day. Where do you ski to find the best glide?	In the shade, It does not matter, In the sun
Q7	For racing skate skis, which ski property or preparation factor has the greatest effect on glide in each snow condition?	Ski camber, Structure, Wax, Hand structure
Q8	For classic racing skis, which ski property or preparation factor has the greatest effect on glide in each snow condition?	Ski camber, Structure, Wax, Hand structure
Q9	You have the best possible skis and waxes. Now choose between 4 different stone grinds. Which one do you select to get the best glide for each snow type?	Very fine, Fine, Coarse, Very coarse
Q10	You have the best possible skis and stone grinds. Now choose between 5 glide waxes of varying hardness. Which one do you select to get the best glide for each snow type?	Very Soft, Soft, Medium, Hard, Very Hard

Questionnaire items were developed iteratively—in Swedish—by the author team in consultation with experienced trainers, skiers, team leaders, and XC waxing practitioners, including personnel from the Swedish Olympic Committee, the waxing and ski service teams of the Swedish Ski Association (SSF), and the Swedish Biathlon Association (SSSF). This expert input informed the study design, item selection, and wording. Items were subsequently pilot checked to confirm terminology and response-option clarity. For the present article, the original Swedish questions and answer options were translated into English; the wording shown here is the authors’ translation of the Swedish questionnaire used for data collection.

### 2.3 Data analysis

Participant characteristics and questionnaire responses were summarised and stratified by expert group. Descriptive statistics were presented as frequencies and percentages for categorical variables, and medians with interquartile ranges for continuous variables. To quantify the level of consensus among participants, we calculated the agreement coefficient A [39], for each question and snow type combination using the agrmt package in R, [40].

van der Eijk’s agreement coefficient A is designed explicitly for ordered rating data. It ranges from −1 (complete polarisation: responses concentrated at opposite ends) to +1 (complete agreement: responses concentrated in a single category), with 0 indicating a uniform distribution across categories. This measure addresses limitations of traditional agreement indices by accounting for the number of response categories and avoiding the assumption of uniform distributions [39]. Agreement was calculated separately for the overall sample, the expert group, and the non-expert group.

To compare responses between expert and non-expert groups, we calculated Cliff’s δ, a non-parametric effect size measure appropriate for ordinal data [37]. Cliff’s δ ranges from −1 to +1, where values near 0 indicate no difference between groups, and values approaching ±1 indicate complete separation between groups. Effect sizes were interpreted using cut-offs based on Vargha and Delaney [38], converted to Cliff’s δ using the relationship δ=2A−1. The resulting thresholds were: negligible (|δ|<0.11), small (|δ|≥0.11), medium (|δ|≥0.28), and large (|δ|≥0.43). These cutoffs correspond to Vargha-Delaney A values of 0.56, 0.64, and 0.71, respectively.

To address unbalanced sample sizes and within-participant dependencies, we applied a stratified bootstrap procedure. Resampling was performed at the participant level, with 10000 bootstrap iterations. Resampling was stratified by expert group to maintain the original proportion of experts and non-experts in each bootstrap sample. For each bootstrap sample, we calculated agreement coefficients (overall, expert, and non-expert) and Cliff’s δ for all question-snow type combinations. Ninety-five per cent confidence intervals were calculated as the 2.5th and 97.5th percentiles of the bootstrap distribution. To assess the statistical significance of group differences, we determined the proportion of bootstrap samples in which Cliff’s δ exceeded the threshold for a negligible effect size (|δ|>0.11). Differences were considered statistically meaningful when more than 95% of bootstrap samples showed |δ|>0.11 in a consistent direction, representing a minimal importance difference approach.

For question-level and snow type-level analyses, we averaged the metrics across the relevant dimensions within each bootstrap sample before calculating confidence intervals, thereby preserving the correlation structure in the data. A fixed random seed (4987) was used to ensure reproducibility of all bootstrap procedures. All statistical analyses were performed in R version 4.4.0 [41].

## 3 Results

### 3.1 Participants characteristics

A total of 249 participants completed the questionnaire. The sample consisted predominantly of middle-aged males (40–59 years, *n* = 149, 60%). Regarding activity levels, the majority of respondents reported low frequencies of ski preparation (<100 per year) and moderate annual skiing distances (100–1,000 km). The expert group comprised 20 participants (8%), who were distinguished by high preparation volume and greater annual skiing distance. Detailed demographic characteristics stratified by group are summarised in Table 3.

**Table 3:** Respondent characteristics for the full sample, experts, and non-experts.

Respondent characteristics	Full sample		Experts		Non-experts	
	(*n* = 249)		(*n* = 20)		(*n* = 229)	
Sex						
Female	14	(6%)	1	(5%)	13	(6%)
Male	234	(94%)	19	(95%)	215	(94%)
Age						
≤29	36	(14%)	1	(5%)	35	(15%)
30–39	23	(9%)	2	(10%)	21	(9%)
40–49	79	(32%)	6	(30%)	73	(32%)
50–59	79	(32%)	9	(45%)	70	(31%)
60–69	24	(10%)	1	(5%)	23	(10%)
≥70	8	(3%)	1	(5%)	7	(3%)
Yearly skiing distance						
<100 km	6	(2%)	0	(0%)	6	(3%)
100–1,000 km	115	(46%)	7	(35%)	108	(47%)
1,000–2,000 km	88	(35%)	9	(45%)	79	(34%)
>2,000 km	40	(16%)	4	(20%)	36	(16%)
Yearly number of ski preparations						
<100	137	(55%)	0	(0%)	137	(60%)
100–500	89	(36%)	0	(0%)	89	(39%)
500–1,000	17	(7%)	15	(75%)	2	(1%)
>1,000	6	(2%)	5	(25%)	1	(0%)
Roles						
Waxer	105	(42%)	20	(100%)	85	(37%)
Elite skier	33	(13%)	2	(10%)	31	(14%)
Coach	33	(13%)	2	(10%)	31	(14%)
Non-elite skier	152	(61%)	6	(30%)	146	(64%)

Percentage within each column and variable.

### 3.2 Descriptive response patterns

**Figure 1:**
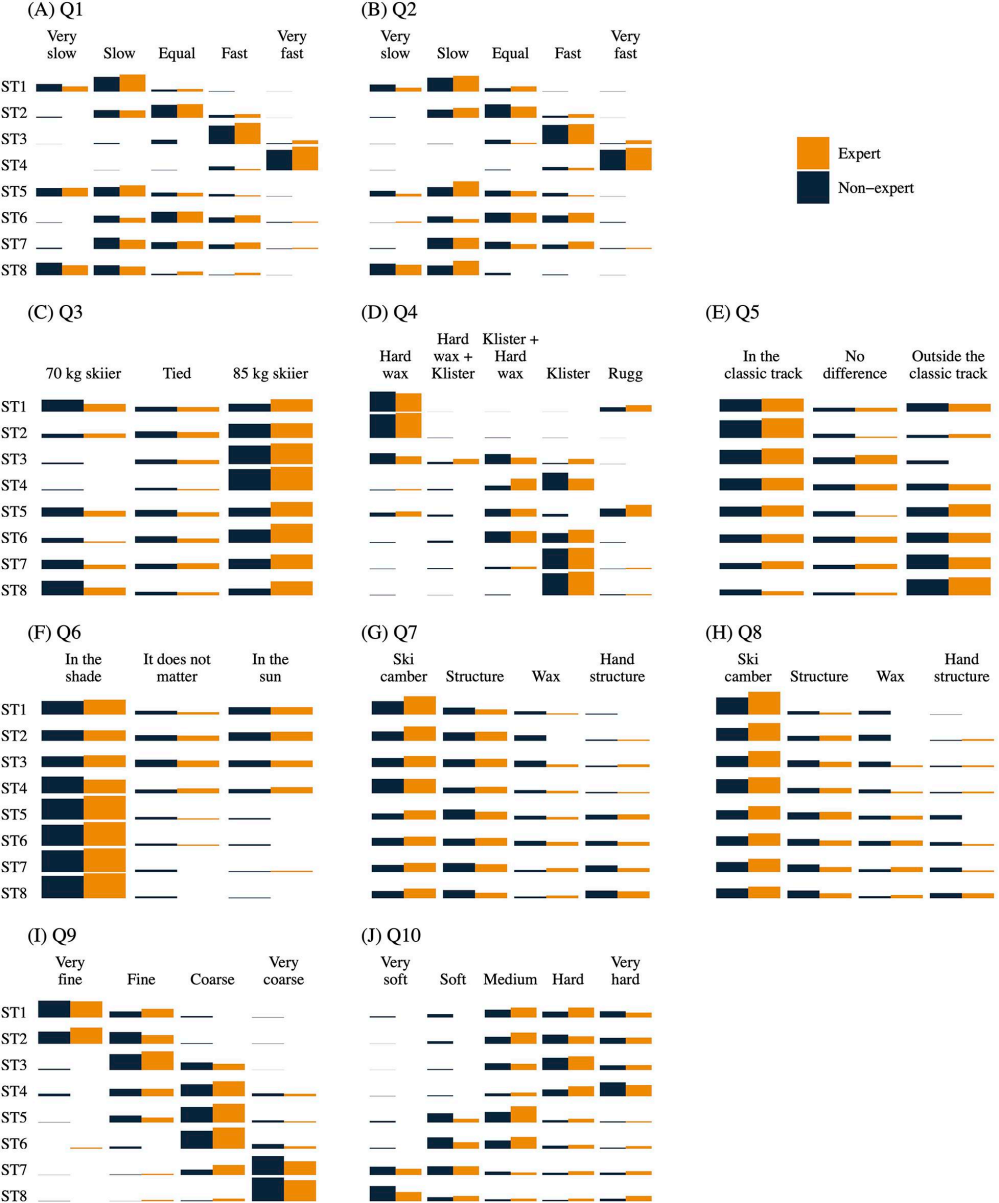
Descriptive response distributions for Q1–Q10 across snow types ST1–ST8, shown separately for experts (orange) and non-experts (dark blue). Panels (A–J) correspond to questionnaire items Q1–Q10 defined in Table 2, and snow-type definitions (ST1–ST8) are given in Table 1. Within each snow type (rows), bar length represents the percentage of respondents in each group selecting a given response option (columns); percentages sum to 100% within each snow type and group (missing responses excluded). Full numerical results are provided in Supplementary Tables S1–S10.

Figure 1 provides an overview of the response distributions across all questions (Q1–Q10) and snow types (ST1–ST8), for experts (orange) and non-experts (black). Detailed numerical summaries are available in Supplementary Tables S1–S10.

### 3.3 Agreement analysis

**Figure 2:**
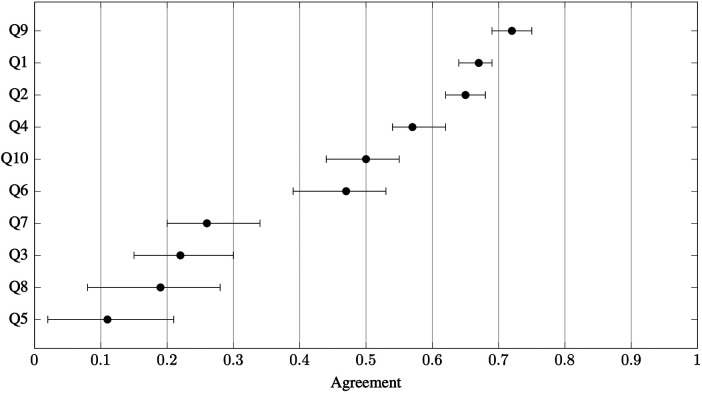
Agreement for the full sample (both experts and non-experts), for each question Q1–Q10, defined in Table 2. Error bars represent 95% bootstrap confidence intervals. van der Eijk’s agreement coefficient ranges from −1 (polarisation) to +1 (consensus), with 0 indicating uniform distribution.

**Figure 3:**
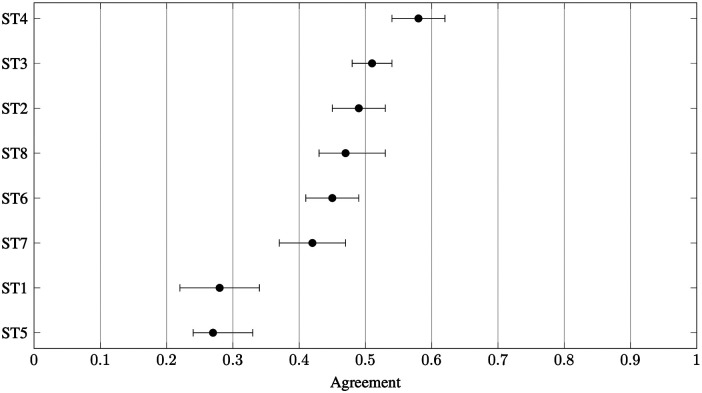
Agreement for the full sample (both experts and non-experts) by snow type ST1–ST8, defined in Table 1. Error bars represent 95% bootstrap confidence intervals. van der Eijk’s agreement coefficient ranges from −1 (polarisation) to +1 (consensus), with 0 indicating uniform distribution.

Agreement for the *full sample* (both experts and non-experts) varied substantially across questions (Q1–Q10) and snow types (ST1–ST8), with stone-grind selection and perceived ski speed generating the highest consensus, whereas positioning and glide-determinant questions revealed considerable divergence in opinion. Figure 2 displays agreement coefficients by question, showing the highest values for stone grind structures (Q9) and perceived ski speed on different snow types (Q1, Q2). The lowest agreement was seen for double-poling positioning (Q5), glide determinants (Q7, Q8), and the paired glide test comparing skier mass (Q9). Figure 3 presents agreement by snow type, revealing that frozen wet snow (ST4) elicited the highest consensus, whereas new snow (ST1) and moist fine-grained snow (ST5) showed the lowest. The specific question–snow type combinations with the highest (A>0.85) and lowest (A≤0.00) agreement are summarised in Table 4. Complete agreement values for all combinations, including subgroup analyses, are provided in Supplementary Table S11.

**Table 4:** Question (Q) and snow type (ST) combinations with the highest (>0.85) and lowest (≤0) agreement. Questions and snow types are defined in Tables 1, 2, respectively.

Question	Snow type	A	95% CI
Highest agreement			
Q4	ST2	0.95	[0.90, 0.98]
Q9	ST8	0.94	[0.89, 0.97]
Q1	ST4	0.90	[0.86, 0.93]
Q4	ST8	0.89	[0.83, 0.94]
Q2	ST4	0.88	[0.83, 0.92]
Q6	ST8	0.88	[0.82, 0.93]
Lowest agreement			
Q7	ST7	0.00	[−0.13, 0.18]
Q6	ST1	−0.04	[−0.32, 0.23]
Q3	ST8	−0.05	[−0.33, 0.22]
Q5	ST6	−0.11	[−0.33, 0.15]
Q8	ST1	−0.11	[−0.35, 0.12]
Q3	ST1	−0.14	[−0.41, 0.14]
Q7	ST8	−0.14	[−0.23, 0.05]
Q6	ST2	−0.15	[−0.39, 0.13]
Q3	ST5	−0.17	[−0.33, 0.11]
Q5	ST1	−0.19	[−0.46, 0.08]
Q3	ST7	−0.20	[−0.43, 0.09]
Q5	ST5	−0.37	[−0.50, −0.06]

A, van der Eijk’s agreement coefficient; ranges from −1 (polarisation) to +1 (consensus), with 0 indicating uniform distribution.

### 3.4 Expert vs. non-expert differences

Expert and non-expert respondents differed most notably in their views on glide determinants. Experts more frequently identified camber as the primary glide determinant, both for skate skiing (Q7; 48% vs. 38%, δ=−0.41, 95% CI [−0.56,−0.26]) and classic skiing (Q8; 61% vs. 47%, δ=−0.31, 95% CI [−0.50,−0.11]). Experts also more often preferred skiing in shadowed snow on sunny days for best glide (Q6; 73% vs. 69%, δ=−0.28, 95% CI [−0.38,−0.18]) and double-poling in the tracks (47% vs. 44%, δ=−0.23, 95% CI [−0.31,−0.14]). Full results are provided in Supplementary Tables S11–S12.

## 4 Discussion

This study investigated perceptions of ski preparation and glide characteristics within the Swedish ski community. The findings revealed that, although there is a shared baseline of knowledge regarding ski structure and speed perception, significant divergences exist regarding the fundamental determinants of glide. Expert ski technicians demonstrated higher within-group consensus than non-experts for perceived ski speed in skate skiing and glide characteristics in moist transformed snow, and they consistently placed greater emphasis on ski camber as a key determinant of performance. Taken together, these patterns suggest that expertise is associated with both greater consensus and distinct priorities regarding the ski–snow system. The present findings characterise practitioners’ perceptions and decision-making heuristics, rather than directly validating physical mechanisms or measuring objective performance outcomes.

### 4.1 Interpreting agreement levels

The agreement analysis showed that consensus was highest for structure selection and for perceived ski speed across snow types, whereas it was lowest for glide determinants, paired glide tests involving skier mass, and positioning during double poling. Moreover, frozen wet snow (ST4) elicited the highest agreement, while new snow (ST1) and moist fine-grained snow (ST5) showed the lowest levels of consensus.

These patterns are consistent with known differences in the physical stability of the underlying snow regimes. Frozen, coarse-grained and refrozen wet snow typically exhibits comparatively stable grain morphology and liquid-water content over the course of a race, creating a tribological environment in which established structural solutions tend to perform reliably across skiers and setups [18, 20–22, 29]. In contrast, new snow is thermodynamically less stable and undergoes relatively rapid metamorphism, including crystal rounding and changes in surface roughness, particularly near the melting point [29, 32]. Moist fine-grained snow similarly occupies an intermediate regime in which small variations in temperature, contamination, or traffic can alter water-film formation and contact mechanics in ways that are difficult to predict [11, 19].

From this perspective, high agreement in frozen wet snow likely reflects that technicians and skiers repeatedly encounter similar, physically stable conditions in which the same structural and waxing strategies tend to work. Conversely, the low agreement in new and moist fine-grained snow suggests that practitioners are confronted with more variable and less tractable conditions, which naturally foster a wider diversity of heuristics and preparation philosophies. This interpretation aligns with broader work on decision-making under uncertainty, in which less predictable environments are associated with greater reliance on idiosyncratic rules-of-thumb and higher between-person variability [33, 34].

### 4.2 The expert gap: mechanical properties vs. chemical preparation

The most profound divergence between experts and non-experts concerned the prioritisation of glide determinants. Experts consistently ranked *mechanical properties*, particularly the ski camber, above *chemical preparation* (waxes and additives). This aligns with tribological evidence showing that stiffness profiles govern the apparent contact area, pressure distribution, and load partitioning, and thereby dominate snow deformation, water-film generation, and the effective coefficient of friction [5, 20–23, 26, 27]. Non-experts, by contrast, placed less weight on camber, likely because waxing is visible, well-marketed, and easily adapted before skiing, whereas camber is a fixed mechanical property that requires specialised tools and access to many ski pairs to assess properly. In essence, the ski’s mechanical properties set the boundary conditions within which micro-scale interventions, such as waxing and hand structuring, can operate, with load distribution determining the real area of contact and thus the regime in which chemical and textural adjustments are effective [11].

The lower emphasis on camber among non-experts may reflect a relative overvaluation of *chemical preparation* relative to the *mechanical properties* of the ski. Wax choice and application are highly visible, frequently marketed, and can be adjusted shortly before competition, whereas camber selection is constrained by the available number of skis and requires more specialised measurement and testing. For recreational skiers working with a small number of ski pairs, it is therefore plausible that waxing appears as the dominant lever to influence glide, even though the underlying mechanics indicate that camber and load distribution are primary drivers of frictional behaviour.

This “expert gap” in the perceived hierarchy of glide determinants has practical implications. First, it suggests that education aimed at non-experts could profitably emphasise the foundational role of ski mechanics, e.g., by illustrating how camber mismatch can negate the benefits of otherwise optimal wax and structure choices. Second, it underscores the value of systematic ski-fleet management and camber profiling at higher performance levels, where technicians routinely handle many pairs of skis and can exploit mechanical optimisation more fully. Finally, recognising that experts and non-experts may talk past each other when using the same vocabulary (e.g., “fast skis”) but prioritising different underlying factors could improve communication between elite service teams and the broader skiing community.

### 4.3 Limitations

This study has several limitations. First, the sample relied on voluntary participation and may over-represent skiers with above-average interest in ski preparation, potentially inflating consensus levels. Second, the expert subgroup was necessarily small (*n* = 20) to ensure validity as elite ski-technicians constitute a limited population. The small group size ensures validity but does limit the precision of estimates. Third, all responses were self-reported and represent subjective perceptions rather than direct physical measurements of glide performance. Fourth, the questionnaire format did not allow participants to seek clarification on ambiguous terminology, which may have introduced response variability. Interviews could have reduced such variability and potentially strengthened observed consensus levels. In addition, several specific sub-topics, such as snow contamination and temperature-dependent structural effects, were omitted to maintain a manageable questionnaire length.

While the reliance on perceptual data is a limitation, gliding properties in competitive settings are still evaluated through field testing and invariably rely on the skier’s subjective judgement [12]. Our findings should therefore be interpreted within the context of a sport in which perceptual assessment remains integral to ski preparation.

### 4.4 Future work

Future research should more directly bridge perception and physics by targeting the topics with lowest agreement (e.g., glide determinants, skier-mass effects in paired glide tests, and double-poling positioning) and combining survey responses with controlled friction measurements and field validation. Longitudinal studies may clarify how practitioners acquire and refine what, in effect, amounts to a form of tribology-based intuition, and whether perceptual consensus changes with accumulated experience. To reduce interpretation bias inherent to questionnaire items, qualitative follow-up (e.g., cognitive interviews/think-aloud protocols) should be used to refine terminology and determine whether response dispersion reflects true disagreement or heterogeneous interpretation. Finally, integrating objective on-snow measurements (e.g., GNSS/IMU-based speed and technique metrics, RTK-based friction estimation, and instrumented skis/poles and tribometer measurements) can provide scalable feedback that complements subjective glide testing and enhances technician training and evidence-based preparation practices for non-experts.

## 5 Conclusions

This study provides the first systematic assessment of perceptual consensus on ski–snow performance within the Swedish skiing community. Regarding the level of agreement (RQ1), consensus varied substantially by topic: agreement was high for practical decisions such as structure selection and perceived ski speed, whereas low agreement characterised more complex variables such as glide determinants and positioning. Furthermore, consensus was strongly linked to snow stability, with frozen wet snow eliciting the highest agreement and new snow the lowest.

Regarding expert consensus (RQ2), expert ski technicians demonstrated higher within-group agreement than non-experts, particularly for specific technical domains, such as perceived ski speed in skate skiing and preparation for moist transformed snow.

Finally, regarding systematic differences (RQ3), a clear divergence emerged in technical prioritisation. Experts consistently identified ski camber as the dominant factor for glide performance, whereas non-experts significantly undervalued this mechanical property in favour of chemical treatments. This suggests that expertise related to ski–snow tribology is defined not only by knowing “what to do” but also by a superior mental model of the hierarchy of physical factors governing ski–snow interaction.

Together, these results contribute to a deeper understanding of practitioners’ reasoning in ski preparation, support communication between recreational and elite environments, and inform future applied tribology research in sport.

## Data Availability

The datasets generated for this study can be found here: https://doi.org/10.6084/m9.figshare.31044988.

## Footnotes

^1^With the FIS fluor ban introduced 2023/2024, this work focuses on how practitioners reason about glide across snow types, independent of specific product chemistries.
