# Correction: Perceptual consensus on cross-country ski–snow performance: a questionnaire study of experts and non-experts

**DOI:** 10.3389/fspor.2026.1808795

**Published:** 2026-03-03

**Authors:** Anton Kalén, Joakim Abrahamsson, Kalle Kalliorinne, Hans-Christer Holmberg, Andreas Almqvist

**Affiliations:** 1 Digital Services and Systems, Department of Computer Science, Electrical and Space Engineering, Luleå University of Technology, Luleå, Sweden; 2 Swedish Olympic Committee, Stockholm, Sweden; 3 Student, Researcher and Educational Support, Professional Services, Luleå University of Technology, Luleå, Sweden; 4 Machine Elements, Department of Engineering Sciences and Mathematics, Luleå University of Technology, Luleå, Sweden; 5 The Swedish Research Centre for Sports and Performance Technology (SPORTC), Luleå University of Technology, Luleå, Sweden

**Keywords:** agreement analysis, expertise, glide performance, perception, ski preparation, skiing, snow, tribology

1000 km was incorrectly written as “100 km”.

A correction has been made to the section 2 Materials and methods, 2.1 Study design and recruitment, Paragraph Number 2:

“A total of 249 participants completed the questionnaire. We categorised participants as experts (*n* = 20, 8%) if they met the following criteria:

• Over 500 ski preparations per year.

• Over 1,000 km of skiing per year.

• Reported a role as waxer.

The remaining participants were classified as non-experts (*n* = 229, 92%). This classification was established *post-hoc* in consultation with national team ski technicians from XC skiing and biathlon to ensure a sufficiently high threshold was established to be considered an expert.”

The original version of this article has been updated.

