# Supplementary material for: Perceptual consensus on cross-country ski–snow performance: a questionnaire study of experts and non-experts
Source: Front Sports Act Living. 2026 Feb 10;8:1766019. doi: 10.3389/fspor.2026.1766019 (PMC12929394; doi:10.3389/fspor.2026.1766019)
Supplement: Supplementary file 1 [file Datasheet1.pdf]

Supplementary Material

1 Descriptive results

Supplementary Tables 1–10 report descriptive response distributions for each questionnaire item (Q1–Q10; see Table 1), stratified by subgroup (*Experts*, *Non-experts*) and for the *Full sample*. For each snow type (ST1–ST8; definitions in Table 2), entries are shown as count, *within-group percentage*, for each response option, where percentages are calculated within subgroup and snow type using non-missing responses. "NA" denotes missing responses and is reported as counts, but the values are excluded from percentage calculations. The rightmost "Total" column aggregates responses across all snow types for the item within each subgroup.

Supplementary Table 1. Q1: In classic skiing, how fast do you perceive the conditions to be for each snow type?

	New snow	Fine-grained snow	Trans-formed snow	Frozen wet snow	Moist fine-grained snow	Moist trans-formed snow	Wet corn snow	Very wet corn snow	Total
**Expert**									
Very slow	4 (21%)	0 (0%)	0 (0%)	0 (0%)	7 (35%)	0 (0%)	0 (0%)	8 (40%)	19 (12%)
Slow	13 (68%)	6 (30%)	0 (0%)	0 (0%)	9 (45%)	4 (20%)	7 (37%)	7 (35%)	46 (29%)
Equal	2 (11%)	11 (55%)	0 (0%)	0 (0%)	3 (15%)	9 (45%)	6 (32%)	3 (15%)	34 (22%)
Fast	0 (0%)	3 (15%)	17 (85%)	1 (5%)	1 (5%)	6 (30%)	5 (26%)	2 (10%)	35 (22%)
Very fast	0 (0%)	0 (0%)	3 (15%)	18 (95%)	0 (0%)	1 (5%)	1 (5%)	0 (0%)	23 (15%)
NA	1	0	0	1	0	0	1	0	3
**Non-expert**									
Very slow	65 (30%)	10 (5%)	1 (0%)	0 (0%)	68 (34%)	4 (2%)	11 (6%)	98 (50%)	257 (16%)
Slow	126 (59%)	64 (31%)	9 (4%)	2 (1%)	76 (38%)	56 (29%)	91 (46%)	78 (40%)	502 (31%)
Equal	18 (8%)	108 (52%)	36 (18%)	3 (1%)	32 (16%)	86 (44%)	55 (28%)	12 (6%)	350 (22%)
Fast	4 (2%)	24 (12%)	151 (74%)	33 (16%)	20 (10%)	42 (22%)	37 (19%)	5 (3%)	316 (19%)
Very fast	1 (0%)	1 (0%)	7 (3%)	172 (82%)	3 (2%)	7 (4%)	5 (3%)	2 (1%)	198 (12%)
NA	15	22	25	19	30	34	30	34	209
**Full sample**									
Very slow	69 (30%)	10 (4%)	1 (0%)	0 (0%)	75 (34%)	4 (2%)	11 (5%)	106 (49%)	276 (16%)
Slow	139 (60%)	70 (31%)	9 (4%)	2 (1%)	85 (39%)	60 (28%)	98 (45%)	85 (40%)	548 (31%)
Equal	20 (9%)	119 (52%)	36 (16%)	3 (1%)	35 (16%)	95 (44%)	61 (28%)	15 (7%)	384 (22%)
Fast	4 (2%)	27 (12%)	168 (75%)	34 (15%)	21 (10%)	48 (22%)	42 (19%)	7 (3%)	351 (20%)
Very fast	1 (0%)	1 (0%)	10 (4%)	190 (83%)	3 (1%)	8 (4%)	6 (3%)	2 (1%)	221 (12%)
NA	16	22	25	20	30	34	31	34	212

Supplementary Table 2. Q2: In skate skiing, how fast do you perceive the conditions to be for each snow type?

	New snow	Fine-grained snow	Trans-formed snow	Frozen wet snow	Moist fine-grained snow	Moist trans-formed snow	Wet corn snow	Very wet corn snow	Total
**Expert**									
Very slow	3 (16%)	0 (0%)	0 (0%)	0 (0%)	2 (11%)	1 (5%)	0 (0%)	8 (42%)	14 (9%)
Slow	12 (63%)	8 (40%)	0 (0%)	0 (0%)	11 (61%)	3 (15%)	9 (45%)	11 (58%)	54 (35%)
Equal	4 (21%)	9 (45%)	1 (5%)	0 (0%)	4 (22%)	8 (40%)	4 (20%)	0 (0%)	30 (19%)
Fast	0 (0%)	3 (15%)	16 (80%)	2 (10%)	1 (6%)	8 (40%)	6 (30%)	0 (0%)	36 (23%)
Very fast	0 (0%)	0 (0%)	3 (15%)	18 (90%)	0 (0%)	0 (0%)	1 (5%)	0 (0%)	22 (14%)
NA	1	0	0	0	2	0	0	1	4
**Non-expert**									
Very slow	54 (28%)	7 (4%)	2 (1%)	2 (1%)	41 (23%)	2 (1%)	7 (4%)	81 (47%)	196 (13%)
Slow	106 (56%)	62 (33%)	3 (2%)	0 (0%)	68 (38%)	46 (26%)	80 (45%)	68 (39%)	433 (30%)
Equal	26 (14%)	102 (54%)	32 (17%)	6 (3%)	43 (24%)	73 (41%)	52 (29%)	17 (10%)	351 (24%)
Fast	2 (1%)	16 (9%)	140 (77%)	25 (13%)	26 (14%)	53 (29%)	32 (18%)	6 (3%)	300 (21%)
Very fast	2 (1%)	1 (1%)	6 (3%)	154 (82%)	2 (1%)	6 (3%)	7 (4%)	2 (1%)	180 (12%)
NA	39	41	46	42	49	49	51	55	372
**Full sample**									
Very slow	57 (27%)	7 (3%)	2 (1%)	2 (1%)	43 (22%)	3 (2%)	7 (4%)	89 (46%)	210 (13%)
Slow	118 (56%)	70 (34%)	3 (1%)	0 (0%)	79 (40%)	49 (24%)	89 (45%)	79 (41%)	487 (30%)
Equal	30 (14%)	111 (53%)	33 (16%)	6 (3%)	47 (24%)	81 (40%)	56 (28%)	17 (9%)	381 (24%)
Fast	2 (1%)	19 (9%)	156 (77%)	27 (13%)	27 (14%)	61 (30%)	38 (19%)	6 (3%)	336 (21%)
Very fast	2 (1%)	1 (0%)	9 (4%)	172 (83%)	2 (1%)	6 (3%)	8 (4%)	2 (1%)	202 (12%)
NA	40	41	46	42	51	49	51	56	376

Supplementary Table 3. Q3: Two skiers have identical skis; one weighs 70~kg and the other 85~kg. In each snow condition, who wins a paired glide test? (Assume aerodynamic drag is negligible.)

	New snow	Fine-grained snow	Trans-formed snow	Frozen wet snow	Moist fine-grained snow	Moist trans-formed snow	Wet corn snow	Very wet corn snow	Total
**Expert**									
70kg skier	5 (31%)	3 (18%)	0 (0%)	0 (0%)	4 (24%)	1 (6%)	3 (18%)	5 (31%)	21 (15%)
Tied	3 (19%)	4 (24%)	3 (17%)	1 (6%)	3 (18%)	3 (18%)	4 (24%)	2 (12%)	23 (17%)
85kg skier	8 (50%)	10 (59%)	15 (83%)	17 (94%)	10 (59%)	13 (76%)	10 (59%)	9 (56%)	92 (68%)
NA	4	3	2	2	3	3	3	4	24
**Non-expert**									
70kg skier	91 (48%)	31 (17%)	12 (6%)	8 (4%)	67 (37%)	36 (20%)	67 (37%)	102 (58%)	414 (28%)
Tied	37 (20%)	49 (26%)	35 (19%)	20 (11%)	49 (27%)	48 (27%)	39 (22%)	25 (14%)	302 (21%)
85kg skier	60 (32%)	105 (57%)	139 (75%)	162 (85%)	65 (36%)	97 (54%)	74 (41%)	48 (27%)	750 (51%)
NA	41	44	43	39	48	48	49	54	366
**Full sample**									
70kg skier	96 (47%)	34 (17%)	12 (6%)	8 (4%)	71 (36%)	37 (19%)	70 (36%)	107 (56%)	435 (27%)
Tied	40 (20%)	53 (26%)	38 (19%)	21 (10%)	52 (26%)	51 (26%)	43 (22%)	27 (14%)	325 (20%)
85kg skier	68 (33%)	115 (57%)	154 (75%)	179 (86%)	75 (38%)	110 (56%)	84 (43%)	57 (30%)	842 (53%)
NA	45	47	45	41	51	51	52	58	390

Supplementary Table 4. Q4: Based on your own experience, which grip system works best on classic skis for each snow type?

	New snow	Fine-grained snow	Trans-formed snow	Frozen wet snow	Moist fine-grained snow	Moist trans-formed snow	Wet corn snow	Very wet corn snow	Total
**Expert**									
Hard wax	14 (70%)	20 (100%)	6 (30%)	1 (5%)	4 (20%)	0 (0%)	0 (0%)	0 (0%)	45 (28%)
Hard wax + klister	0 (0%)	0 (0%)	4 (20%)	0 (0%)	0 (0%)	0 (0%)	0 (0%)	0 (0%)	4 (2%)
Klister + hard wax	0 (0%)	0 (0%)	5 (25%)	9 (45%)	6 (30%)	9 (45%)	2 (10%)	0 (0%)	31 (19%)
Klister	0 (0%)	0 (0%)	4 (20%)	9 (45%)	0 (0%)	10 (50%)	17 (85%)	19 (95%)	59 (37%)
“Rugg”	5 (25%)	0 (0%)	0 (0%)	0 (0%)	9 (45%)	0 (0%)	1 (5%)	1 (5%)	16 (10%)
NA	1 (5%)	0 (0%)	1 (5%)	1 (5%)	1 (5%)	1 (5%)	0 (0%)	0 (0%)	5 (3%)
**Non-expert**									
Hard wax	161 (70%)	187 (82%)	86 (38%)	7 (3%)	31 (14%)	6 (3%)	4 (2%)	4 (2%)	486 (27%)
Hard wax + klister	1 (0%)	3 (1%)	18 (8%)	12 (5%)	16 (7%)	17 (7%)	8 (3%)	2 (1%)	77 (4%)
Klister + hard wax	1 (0%)	4 (2%)	79 (34%)	38 (17%)	59 (26%)	87 (38%)	18 (8%)	7 (3%)	293 (16%)
Klister	1 (0%)	2 (1%)	10 (4%)	140 (61%)	21 (9%)	74 (32%)	160 (70%)	172 (75%)	580 (32%)
“Rugg”	36 (16%)	1 (0%)	2 (1%)	0 (0%)	61 (27%)	5 (2%)	4 (2%)	9 (4%)	118 (6%)
NA	29 (13%)	32 (14%)	34 (15%)	32 (14%)	41 (18%)	40 (17%)	35 (15%)	35 (15%)	278 (15%)
**Full sample**									
Hard wax	175 (70%)	207 (83%)	92 (37%)	8 (3%)	35 (14%)	6 (2%)	4 (2%)	4 (2%)	531 (27%)
Hard wax + klister	1 (0%)	3 (1%)	22 (9%)	12 (5%)	16 (6%)	17 (7%)	8 (3%)	2 (1%)	81 (4%)
Klister + hard wax	1 (0%)	4 (2%)	84 (34%)	47 (19%)	65 (26%)	96 (39%)	20 (8%)	7 (3%)	324 (16%)
Klister	1 (0%)	2 (1%)	14 (6%)	149 (60%)	21 (8%)	84 (34%)	177 (71%)	191 (77%)	639 (32%)
“Rugg”	41 (16%)	1 (0%)	2 (1%)	0 (0%)	70 (28%)	5 (2%)	5 (2%)	10 (4%)	134 (7%)
NA	30 (12%)	32 (13%)	35 (14%)	33 (13%)	42 (17%)	41 (16%)	35 (14%)	35 (14%)	283 (14%)

Supplementary Table 5. Q5: When double poling, where, in each snow condition, should you position yourself on the groomed track to achieve the best glide?

	New snow	Fine-grained snow	Trans-formed snow	Frozen wet snow	Moist fine-grained snow	Moist trans-formed snow	Wet corn snow	Very wet corn snow	Total
**Expert**									
In the classic track	10 (53%)	15 (79%)	12 (63%)	10 (50%)	9 (45%)	7 (35%)	6 (32%)	3 (17%)	72 (47%)
It does not matter	3 (16%)	1 (5%)	7 (37%)	5 (25%)	1 (5%)	5 (25%)	4 (21%)	2 (11%)	28 (18%)
Outside the cl. track	6 (32%)	3 (16%)	0 (0%)	5 (25%)	10 (50%)	8 (40%)	9 (47%)	13 (72%)	54 (35%)
NA	1	1	1	0	0	0	1	2	6
**Non-expert**									
In the classic track	100 (50%)	138 (71%)	110 (57%)	96 (48%)	72 (40%)	62 (34%)	45 (24%)	44 (24%)	667 (44%)
It does not matter	32 (16%)	33 (17%)	53 (28%)	49 (25%)	39 (22%)	46 (25%)	32 (17%)	22 (12%)	306 (20%)
Outside the cl. track	68 (34%)	24 (12%)	29 (15%)	54 (27%)	68 (38%)	75 (41%)	108 (58%)	120 (65%)	546 (36%)
NA	29	34	37	30	50	46	44	43	313
**Full sample**									
In the classic track	110 (50%)	153 (71%)	122 (58%)	106 (48%)	81 (41%)	69 (34%)	51 (25%)	47 (23%)	739 (44%)
It does not matter	35 (16%)	34 (16%)	60 (28%)	54 (25%)	40 (20%)	51 (25%)	36 (18%)	24 (12%)	334 (20%)
Outside the cl. track	74 (34%)	27 (13%)	29 (14%)	59 (27%)	78 (39%)	83 (41%)	117 (57%)	133 (65%)	600 (36%)
NA	30	35	38	30	50	46	45	45	319

Supplementary Table 6. Q6: You are skate skiing on a sunny day. Where do you ski to find the best glide?

	New snow	Fine-grained snow	Trans-formed snow	Frozen wet snow	Moist fine-grained snow	Moist trans-formed snow	Wet corn snow	Very wet corn snow	Total
**Expert**									
In the shade	12 (60%)	8 (42%)	9 (47%)	11 (55%)	19 (95%)	18 (95%)	18 (95%)	18 (100%)	113 (73%)
It does not matter	2 (10%)	4 (21%)	5 (26%)	4 (20%)	1 (5%)	1 (5%)	0 (0%)	0 (0%)	17 (11%)
In the sun	6 (30%)	7 (37%)	5 (26%)	5 (25%)	0 (0%)	0 (0%)	1 (5%)	0 (0%)	24 (16%)
NA	0	1	1	0	0	1	1	2	6
**Non-expert**									
In the shade	99 (55%)	76 (43%)	78 (43%)	125 (67%)	143 (84%)	147 (84%)	153 (87%)	154 (90%)	975 (69%)
It does not matter	28 (15%)	41 (23%)	54 (30%)	29 (16%)	18 (11%)	16 (9%)	17 (10%)	13 (8%)	216 (15%)
In the sun	54 (30%)	61 (34%)	49 (27%)	32 (17%)	10 (6%)	11 (6%)	6 (3%)	5 (3%)	228 (16%)
NA	48	51	48	43	58	55	53	57	413
**Full sample**									
In the shade	111 (55%)	84 (43%)	87 (44%)	136 (66%)	162 (85%)	165 (85%)	171 (88%)	172 (91%)	1088 (69%)
It does not matter	30 (15%)	45 (23%)	59 (30%)	33 (16%)	19 (10%)	17 (9%)	17 (9%)	13 (7%)	233 (15%)
In the sun	60 (30%)	68 (35%)	54 (27%)	37 (18%)	10 (5%)	11 (6%)	7 (4%)	5 (3%)	252 (16%)
NA	48	52	49	43	58	56	54	59	419

Supplementary Table 7. Q7: For racing skate skis, which ski property or preparation factor has the greatest effect on glide in each snow condition?

	New snow	Fine-grained snow	Trans-formed snow	Frozen wet snow	Moist fine-grained snow	Moist trans-formed snow	Wet corn snow	Very wet corn snow	Total
**Expert**									
Ski camber	14 (74%)	11 (58%)	8 (44%)	11 (58%)	7 (37%)	7 (37%)	7 (37%)	7 (39%)	72 (48%)
Structure	4 (21%)	7 (37%)	6 (33%)	5 (26%)	6 (32%)	6 (32%)	6 (32%)	4 (22%)	44 (29%)
Wax	1 (5%)	0 (0%)	2 (11%)	2 (11%)	2 (11%)	3 (16%)	3 (16%)	2 (11%)	15 (10%)
Hand structure	0 (0%)	1 (5%)	2 (11%)	1 (5%)	4 (21%)	3 (16%)	3 (16%)	5 (28%)	19 (13%)
NA	1	1	2	1	1	1	1	2	10
**Non-expert**									
Ski camber	91 (54%)	62 (38%)	59 (36%)	93 (58%)	40 (25%)	51 (32%)	47 (29%)	48 (29%)	491 (38%)
Structure	48 (28%)	56 (34%)	51 (31%)	37 (23%)	66 (40%)	46 (29%)	59 (36%)	54 (33%)	417 (32%)
Wax	23 (14%)	37 (23%)	42 (26%)	21 (13%)	25 (15%)	32 (20%)	14 (9%)	12 (7%)	206 (16%)
Hand structure	7 (4%)	9 (5%)	10 (6%)	9 (6%)	32 (20%)	31 (19%)	44 (27%)	51 (31%)	193 (15%)
NA	60	65	67	69	66	69	65	64	525
**Full sample**									
Ski camber	105 (56%)	73 (40%)	67 (37%)	104 (58%)	47 (26%)	58 (32%)	54 (30%)	55 (30%)	563 (39%)
Structure	52 (28%)	63 (34%)	57 (32%)	42 (23%)	72 (40%)	52 (29%)	65 (36%)	58 (32%)	461 (32%)
Wax	24 (13%)	37 (20%)	44 (24%)	23 (13%)	27 (15%)	35 (20%)	17 (9%)	14 (8%)	221 (15%)
Hand structure	7 (4%)	10 (5%)	12 (7%)	10 (6%)	36 (20%)	34 (19%)	47 (26%)	56 (31%)	212 (15%)
NA	61	66	69	70	67	70	66	66	535

Supplementary Table 8. Q8: For racing classic skis, which ski property or preparation factor has the greatest effect on glide in each snow condition?

	New snow	Fine-grained snow	Trans-formed snow	Frozen wet snow	Moist fine-grained snow	Moist trans-formed snow	Wet corn snow	Very wet corn snow	Total
**Expert**									
Ski camber	11 (92%)	10 (71%)	9 (64%)	9 (64%)	7 (54%)	7 (50%)	8 (53%)	7 (47%)	68 (61%)
Structure	1 (8%)	3 (21%)	3 (21%)	3 (21%)	4 (31%)	3 (21%)	3 (20%)	3 (20%)	23 (21%)
Wax	0 (0%)	0 (0%)	1 (7%)	1 (7%)	2 (15%)	3 (21%)	3 (20%)	2 (13%)	12 (11%)
Hand structure	0 (0%)	1 (7%)	1 (7%)	1	0 (0%)	1 (7%)	1 (7%)	3 (20%)	8 (7%)
NA	8	6	6	6	7	6	5	5	49
**Non-expert**									
Ski camber	93 (69%)	78 (52%)	67 (45%)	79 (7%)	53 (38%)	59 (38%)	64 (40%)	65 (40%)	558 (47%)
Structure	19 (14%)	30 (20%)	41 (27%)	30 (57%)	41 (29%)	42 (27%)	49 (31%)	51 (31%)	303 (25%)
Wax	21 (16%)	37 (25%)	33 (22%)	22 (22%)	22 (16%)	30 (19%)	18 (11%)	14 (9%)	197 (16%)
Hand structure	2 (1%)	6 (4%)	9 (6%)	8 (16%)	25 (18%)	23 (15%)	29 (18%)	34 (21%)	136 (11%)
NA	94	78	79	90 (6%)	88	75	69	65	638
**Full sample**									
Ski camber	104 (71%)	88 (53%)	76 (46%)	88 (58%)	60 (39%)	66 (39%)	72 (41%)	72 (40%)	626 (48%)
Structure	20 (14%)	33 (20%)	44 (27%)	33 (22%)	45 (29%)	45 (27%)	52 (30%)	54 (30%)	326 (25%)
Wax	21 (14%)	37 (22%)	34 (21%)	23 (15%)	24 (16%)	33 (20%)	21 (12%)	16 (9%)	209 (16%)
Hand structure	2 (1%)	7 (4%)	10 (6%)	9 (6%)	25 (16%)	24 (14%)	30 (17%)	37 (21%)	144 (11%)
NA	102	84	85	96	95	81	74	70	687

Supplementary Table 9. Q9: You have the best possible skis and waxes. Now choose between 4 different stone grinds. Which one do you select to get the best glide for each snow type?

	New snow	Fine-grained snow	Trans-formed snow	Frozen wet snow	Moist fine-grained snow	Moist trans-formed snow	Wet corn snow	Very wet corn snow	Total
**Expert**									
Very fine	13 (65%)	13 (65%)	0 (0%)	0 (0%)	0 (0%)	1 (5%)	0 (0%)	0 (0%)	27 (17%)
Fine	7 (35%)	7 (35%)	15 (75%)	6 (30%)	4 (20%)	0 (0%)	1 (5%)	1 (5%)	41 (26%)
Coarse	0 (0%)	0 (0%)	5 (25%)	12 (60%)	15 (75%)	17 (85%)	8 (40%)	2 (10%)	59 (37%)
Very coarse	0 (0%)	0 (0%)	0 (0%)	2 (10%)	1 (5%)	2 (10%)	11 (55%)	16 (80%)	32 (20%)
NA	0 (0%)	0 (0%)	0 (0%)	0 (0%)	0 (0%)	0 (0%)	0 (0%)	1 (5%)	1 (1%)
**Non-expert**									
Very fine	121 (53%)	86 (38%)	9 (4%)	18 (8%)	2 (1%)	0 (0%)	1 (0%)	2 (1%)	239 (13%)
Fine	44 (19%)	83 (36%)	109 (48%)	51 (22%)	48 (21%)	16 (7%)	4 (2%)	1 (0%)	356 (19%)
Coarse	11 (5%)	5 (2%)	52 (23%)	82 (36%)	107 (47%)	126 (55%)	38 (17%)	6 (3%)	427 (23%)
Very coarse	3 (1%)	2 (1%)	1 (0%)	20 (9%)	15 (7%)	33 (14%)	134 (59%)	167 (73%)	375 (20%)
NA	50 (22%)	53 (23%)	58 (25%)	58 (25%)	57 (25%)	54 (24%)	52 (23%)	53 (23%)	435 (24%)
**Full sample**									
Very fine	134 (54%)	99 (40%)	9 (4%)	18 (7%)	2 (1%)	1 (0%)	1 (0%)	2 (1%)	266 (13%)
Fine	51 (20%)	90 (36%)	124 (50%)	57 (23%)	52 (21%)	16 (6%)	5 (2%)	2 (1%)	397 (20%)
Coarse	11 (4%)	5 (2%)	57 (23%)	94 (38%)	122 (49%)	143 (57%)	46 (18%)	8 (3%)	486 (24%)
Very coarse	3 (1%)	2 (1%)	1 (0%)	22 (9%)	16 (6%)	35 (14%)	145 (58%)	183 (73%)	407 (20%)
NA	50 (20%)	53 (21%)	58 (23%)	58 (23%)	57 (23%)	54 (22%)	52 (21%)	54 (22%)	436 (22%)

Supplementary Table 10. Q10: You have the best possible skis and stone grinds. Now choose between 5 glide waxes of varying hardness. Which one do you select to get the best glide for each snow type?

	New snow	Fine-grained snow	Trans-formed snow	Frozen wet snow	Moist fine-grained snow	Moist trans-formed snow	Wet corn snow	Very wet corn snow	Total
**Expert**									
Very soft	0 (0%)	0 (0%)	0 (0%)	0 (0%)	0 (0%)	0 (0%)	5 (25%)	7 (35%)	12 (8%)
Soft	0 (0%)	0 (0%)	0 (0%)	0 (0%)	3 (15%)	5 (25%)	7 (35%)	4 (20%)	19 (12%)
Medium	8 (40%)	9 (45%)	5 (25%)	3 (15%)	13 (65%)	9 (45%)	2 (10%)	2 (10%)	51 (32%)
Hard	8 (40%)	6 (30%)	11 (55%)	8 (40%)	3 (15%)	3 (15%)	3 (15%)	2 (10%)	44 (28%)
Very hard	4 (20%)	5 (25%)	4 (20%)	9 (45%)	1 (5%)	2 (10%)	3 (15%)	4 (20%)	32 (20%)
NA	0 (0%)	0 (0%)	0 (0%)	0 (0%)	0 (0%)	1 (5%)	0 (0%)	1 (5%)	2 (1%)
**Non-expert**									
Very soft	9 (4%)	1 (0%)	1 (0%)	2 (1%)	11 (5%)	7 (3%)	56 (24%)	102 (45%)	189 (10%)
Soft	24 (10%)	18 (8%)	7 (3%)	7 (3%)	63 (28%)	77 (34%)	59 (26%)	27 (12%)	282 (15%)
Medium	54 (24%)	48 (21%)	46 (20%)	20 (9%)	72 (31%)	56 (24%)	23 (10%)	13 (6%)	332 (18%)
Hard	41 (18%)	63 (28%)	83 (36%)	47 (21%)	16 (7%)	21 (9%)	15 (7%)	9 (4%)	295 (16%)
Very hard	43 (19%)	41 (18%)	32 (14%)	96 (42%)	8 (3%)	8 (3%)	16 (7%)	17 (7%)	261 (14%)
NA	58 (25%)	58 (25%)	60 (26%)	57 (25%)	59 (26%)	60 (26%)	60 (26%)	61 (27%)	473 (26%)
**Full sample**									
Very soft	9 (4%)	1 (0%)	1 (0%)	2 (1%)	11 (4%)	7 (3%)	61 (24%)	109 (44%)	201 (10%)
Soft	24 (10%)	18 (7%)	7 (3%)	7 (3%)	66 (27%)	82 (33%)	66 (27%)	31 (12%)	301 (15%)
Medium	62 (25%)	57 (23%)	51 (20%)	23 (9%)	85 (34%)	65 (26%)	25 (10%)	15 (6%)	383 (19%)
Hard	49 (20%)	69 (28%)	94 (38%)	55 (22%)	19 (8%)	24 (10%)	18 (7%)	11 (4%)	339 (17%)
Very hard	47 (19%)	46 (18%)	36 (14%)	105 (42%)	9 (4%)	10 (4%)	19 (8%)	21 (8%)	293 (15%)
NA	58 (23%)	58 (23%)	60 (24%)	57 (23%)	59 (24%)	61 (24%)	60 (24%)	62 (25%)	475 (24%)

2 Agreement and group differences

Supplementary Table 11. Agreement and expert–non-expert differences by question (Q1–Q10; see Table 2) and snow type (ST1–ST8; see Table 1). Agreement is quantified using van der Eijk's measure of agreement (A) for ordered rating [39], with A ranging from −1 (complete polarisation: responses concentrated at opposite ends) to +1 (complete agreement: responses concentrated in a single category), with 0 indicating no net agreement across categories. Agreement is reported for the Full sample, Experts, and Non-experts, and values are reported as the median (Med) with a 95% bootstrap confidence interval (CI; 10,000 resamples), computed separately for each group. Group-difference agreement is defined as $A_{\text{expert}} - A_{\text{non-expert}}$, with positive values indicating higher agreement among experts. Cliff's delta (δ) quantifies expert–non-expert differences in ordinal responses. A positive δ indicates higher-ranked responses among experts, and a negative δ indicates higher-ranked responses among non-experts. The *Average* is the unweighted mean that summarizes results across snow types within each question. Boldfaced values indicate group-difference estimates with 95% CIs that exclude zero.

	Agreement: Full sample		Agreement: Experts		Agreement: Non-experts		Agreement: Group difference		Cliff's delta	
	Med	95% CI	Med	95% CI	Med	95% CI	Med	95% CI	Med	95% CI
Q1: In classic skiing, how fast do you perceive the conditions to be for each snow type?										
New snow	0.73	[0.68, 0.78]	0.79	[0.64, 0.94]	0.73	[0.67, 0.78]	0.06	[-0.09, 0.22]	-0.04	[-0.26, 0.17]
Fine grained snow	0.65	[0.59, 0.71]	0.70	[0.56, 0.87]	0.65	[0.59, 0.71]	0.05	[-0.10, 0.23]	-0.02	[-0.24, 0.20]
Transformed snow	0.81	[0.75, 0.86]	0.93	[0.83, 1.00]	0.81	[0.75, 0.86]	**0.12**	**[0.01, 0.20]**	0.17	[0.04, 0.31]
Frozen wet snow	0.90	[0.86, 0.93]	0.97	[0.91, 1.00]	0.89	[0.85, 0.92]	**0.08**	**[0.01, 0.14]**	-0.02	[-0.18, 0.11]
Moist fine-grained snow	0.50	[0.43, 0.57]	0.60	[0.42, 0.80]	0.49	[0.42, 0.56]	0.11	[-0.08, 0.32]	-0.19	[-0.40, 0.03]
Moist transformed snow	0.54	[0.48, 0.61]	0.58	[0.40, 0.78]	0.55	[0.48, 0.62]	0.03	[-0.15, 0.25]	-0.02	[-0.23, 0.19]
Wet corn snow	0.54	[0.47, 0.61]	0.50	[0.30, 0.73]	0.54	[0.47, 0.62]	-0.04	[-0.25, 0.20]	-0.02	[-0.26, 0.21]
Very wet corn snow	0.67	[0.61, 0.72]	0.53	[0.27, 0.74]	0.68	[0.62, 0.73]	-0.15	[-0.41, 0.07]	-0.01	[-0.24, 0.22]
Q1 Average	0.67	[0.64, 0.69]	0.70	[0.64, 0.78]	0.66	[0.64, 0.69]	0.06	[-0.03, 0.12]	-0.02	[-0.09, 0.05]
Q2: In skate skiing, how fast do you perceive the conditions to be for each snow type?										
New snow	0.69	[0.63, 0.74]	0.74	[0.59, 0.91]	0.68	[0.62, 0.74]	0.05	[-0.10, 0.24]	-0.10	[-0.31, 0.11]
Fine grained snow	0.68	[0.62, 0.73]	0.65	[0.53, 0.82]	0.68	[0.62, 0.74]	-0.03	[-0.16, 0.15]	-0.16	[-0.38, 0.06]
Transformed snow	0.83	[0.78, 0.88]	0.88	[0.75, 0.98]	0.83	[0.78, 0.88]	0.04	[-0.10, 0.16]	-0.01	[-0.16, 0.15]
Frozen wet snow	0.88	[0.83, 0.92]	0.95	[0.88, 1.00]	0.87	[0.82, 0.92]	0.08	[-0.01, 0.16]	-0.12	[-0.25, 0.00]
Moist fine-grained snow	0.44	[0.37, 0.52]	0.69	[0.50, 0.90]	0.41	[0.34, 0.5]	**0.28**	**[0.06, 0.50]**	**-0.32**	**[-0.51, -0.13]**
Moist transformed snow	0.53	[0.47, 0.59]	0.58	[0.40, 0.78]	0.53	[0.46, 0.6]	0.05	[-0.14, 0.26]	-0.16	[-0.36, 0.05]
Wet corn snow	0.54	[0.46, 0.61]	0.46	[0.24, 0.73]	0.54	[0.46, 0.62]	-0.08	[-0.31, 0.20]	-0.14	[-0.35, 0.08]
Very wet corn snow	0.64	[0.59, 0.70]	0.79	[0.75, 0.9]	0.63	[0.57, 0.69]	**0.16**	**[0.08, 0.28]**	**-0.31**	**[-0.49, -0.13]**
Q2 Average	0.65	[0.62, 0.68]	0.72	[0.69, 0.79]	0.65	[0.62, 0.68]	0.07	[0.03, 0.15]	-0.16	[-0.24, -0.09]
Q3: Two skiers have identical skis; one weighs 70 kg and the other 85 kg. In each snow condition, who wins a paired glide test? (Assume aerodynamic drag is negligible.)										
New snow	-0.14	[-0.41, 0.14]	-0.06	[-0.71, 0.6]	-0.08	[-0.37, 0.2]	0.02	[-0.68, 0.77]	-0.21	[-0.46, 0.05]

Fine grained snow	0.40	[0.29, 0.50]	0.41	[-0.31, 0.76]	0.40	[0.29, 0.51]	0.01	[-0.71, 0.38]	-0.30	[-0.54, -0.07]
Transformed snow	0.70	[0.62, 0.77]	0.83	[0.65, 1.00]	0.68	[0.6, 0.77]	0.15	[-0.06, 0.33]	-0.20	[-0.39, -0.02]
Frozen wet snow	0.82	[0.75, 0.88]	0.94	[0.81, 1.00]	0.81	[0.74, 0.88]	0.13	[-0.01, 0.25]	-0.18	[-0.35, -0.04]
Moist fine-grained snow	-0.17	[-0.33, 0.11]	0.24	[-0.59, 0.73]	-0.17	[-0.31, 0.12]	0.40	[-0.53, 0.88]	-0.17	[-0.42, 0.07]
Moist transformed snow	0.37	[0.24, 0.47]	0.71	[0.31, 0.95]	0.34	[0.19, 0.45]	0.37	[-0.03, 0.66]	-0.16	[-0.39, 0.05]
Wet corn snow	-0.20	[-0.43, 0.09]	0.41	[-0.31, 0.77]	-0.27	[-0.46, 0.04]	0.68	[-0.12, 1.07]	-0.18	[-0.41, 0.05]
Very wet corn snow	-0.05	[-0.33, 0.22]	-0.13	[-0.82, 0.67]	0.05	[-0.24, 0.32]	-0.17	[-0.90, 0.67]	-0.20	[-0.44, 0.06]
Q3 Average	0.22	[0.15, 0.30]	0.42	[0.10, 0.70]	0.22	[0.16, 0.31]	0.20	[-0.14, 0.47]	-0.20	[-0.38, -0.02]
Q4: Based on your own experience, which grip system works best on classic skis for each snow type?										
New snow	0.25	[0.04, 0.45]	-0.05	[-0.87, 0.71]	0.28	[0.06, 0.48]	-0.33	[-1.15, 0.45]	-0.11	[-0.30, 0.10]
Fine grained snow	0.95	[0.90, 0.98]	1.00	[1.00, 1.00]	0.94	[0.9, 0.98]	**0.06**	**[0.02, 0.10]**	**-0.18**	**[-0.24, -0.14]**
Transformed snow	0.25	[0.19, 0.36]	0.28	[0.08, 0.59]	0.24	[0.19, 0.36]	0.04	[-0.20, 0.35]	-0.08	[-0.33, 0.17]
Frozen wet snow	0.78	[0.72, 0.83]	0.65	[0.44, 0.86]	0.79	[0.73, 0.84]	-0.14	[-0.35, 0.08]	**-0.35**	**[-0.55, -0.15]**
Moist fine-grained snow	0.01	[-0.06, 0.13]	0.02	[-0.44, 0.45]	0.01	[-0.05, 0.15]	0.01	[-0.49, 0.42]	-0.15	[-0.40, 0.09]
Moist transformed snow	0.63	[0.57, 0.69]	0.76	[0.75, 0.88]	0.61	[0.55, 0.68]	**0.15**	**[0.10, 0.28]**	-0.10	[-0.30, 0.10]
Wet corn snow	0.84	[0.79, 0.90]	0.90	[0.79, 1.00]	0.84	[0.78, 0.9]	0.06	[-0.07, 0.18]	-0.08	[-0.24, 0.06]
Very wet corn snow	0.89	[0.83, 0.94]	0.98	[0.92, 1.00]	0.88	[0.81, 0.93]	**0.10**	**[0.02, 0.18]**	-0.10	[-0.19, 0.01]
Q4 Average	0.57	[0.54, 0.62]	0.57	[0.44, 0.74]	0.57	[0.54, 0.62]	-0.01	[-0.15, 0.17]	-0.14	[-0.23, -0.06]
Q5: When double poling, where, in each snow condition, should you position yourself on the groomed track to achieve the best glide?										
New snow	-0.19	[-0.46, 0.08]	-0.11	[-0.72, 0.57]	-0.20	[-0.48, 0.09]	0.09	[-0.60, 0.84]	-0.20	[-0.42, 0.04]
Fine grained snow	0.59	[0.45, 0.68]	0.42	[-0.33, 0.94]	0.58	[0.46, 0.68]	-0.16	[-0.94, 0.37]	-0.24	[-0.42, -0.03]
Transformed snow	0.44	[0.34, 0.54]	0.63	[0.50, 0.85]	0.42	[0.31, 0.52]	**0.21**	**[0.04, 0.45]**	**-0.30**	**[-0.47, -0.11]**
Frozen wet snow	0.17	[-0.09, 0.32]	0.25	[-0.50, 0.60]	0.16	[-0.11, 0.32]	0.09	[-0.67, 0.53]	-0.15	[-0.37, 0.07]
Moist fine-grained snow	-0.37	[-0.50, -0.06]	-0.75	[-1.00, 0.22]	-0.30	[-0.46, 0.03]	-0.45	[-0.80, 0.50]	-0.19	[-0.40, 0.04]
Moist transformed snow	-0.11	[-0.33, 0.15]	-0.15	[-0.59, 0.44]	-0.10	[-0.34, 0.16]	-0.05	[-0.56, 0.62]	-0.21	[-0.42, 0.00]
Wet corn snow	0.18	[-0.08, 0.40]	-0.05	[-0.65, 0.53]	0.20	[-0.07, 0.43]	-0.25	[-0.90, 0.41]	**-0.32**	**[-0.53, -0.11]**
Very wet corn snow	0.20	[-0.05, 0.43]	0.44	[-0.36, 0.87]	0.17	[-0.09, 0.42]	0.27	[-0.56, 0.79]	-0.21	[-0.42, -0.01]
Q5 Average	0.11	[0.02, 0.21]	0.09	[-0.18, 0.41]	0.12	[0.03, 0.22]	-0.03	[-0.32, 0.31]	**-0.23**	**[-0.31, -0.14]**
Q6: You are skate skiing on a sunny day. Where do you ski to find the best glide?										
New snow	-0.04	[-0.32, 0.23]	-0.10	[-0.79, 0.63]	-0.04	[-0.34, 0.24]	-0.06	[-0.80, 0.75]	-0.24	[-0.43, -0.03]
Fine grained snow	-0.15	[-0.39, 0.13]	-0.26	[-0.69, 0.45]	-0.14	[-0.39, 0.15]	-0.12	[-0.62, 0.66]	-0.25	[-0.47, -0.03]
Transformed snow	0.17	[-0.07, 0.28]	0.21	[-0.5, 0.57]	0.16	[-0.08, 0.28]	0.05	[-0.67, 0.47]	-0.28	[-0.49, -0.05]
Frozen wet snow	0.44	[0.20, 0.58]	0.20	[-0.55, 0.65]	0.47	[0.22, 0.6]	-0.27	[-1.03, 0.25]	-0.08	[-0.29, 0.12]
Moist fine-grained snow	0.80	[0.72, 0.87]	0.95	[0.83, 1.00]	0.78	[0.69, 0.86]	**0.17**	**[0.03, 0.29]**	**-0.34**	**[-0.43, -0.24]**
Moist transformed snow	0.80	[0.70, 0.87]	0.95	[0.82, 1.00]	0.78	[0.67, 0.86]	**0.17**	**[0.02, 0.30]**	**-0.36**	**[-0.47, -0.24]**
Wet corn snow	0.84	[0.77, 0.90]	0.79	[0.29, 1.00]	0.84	[0.77, 0.9]	-0.05	[-0.54, 0.22]	**-0.32**	**[-0.44, -0.19]**
Very wet corn snow	0.88	[0.82, 0.93]	1.00	[1.00, 1.00]	0.87	[0.8, 0.93]	**0.13**	**[0.07, 0.20]**	**-0.40**	**[-0.51, -0.30]**

Q6 Average	0.47	[0.39, 0.53]	0.47	[0.28, 0.69]	0.46	[0.38, 0.53]	0.00	[-0.19, 0.24]	**-0.28**	**[-0.38, -0.18]**
Q7: For racing skate skis, which ski property or preparation factor has the greatest effect on glide in each snow condition?										
New snow	0.57	[0.49, 0.65]	0.79	[0.59, 0.95]	0.55	[0.46, 0.63]	**0.24**	**[0.02, 0.43]**	**-0.46**	**[-0.61, -0.29]**
Fine grained snow	0.39	[0.31, 0.48]	0.60	[0.24, 0.86]	0.36	[0.28, 0.46]	0.23	[-0.13, 0.50]	**-0.51**	**[-0.66, -0.33]**
Transformed snow	0.33	[0.24, 0.42]	0.41	[-0.06, 0.71]	0.32	[0.21, 0.42]	0.09	[-0.38, 0.40]	**-0.42**	**[-0.62, -0.21]**
Frozen wet snow	0.56	[0.47, 0.65]	0.58	[0.22, 0.82]	0.56	[0.46, 0.65]	0.02	[-0.35, 0.28]	**-0.34**	**[-0.52, -0.15]**
Moist fine-grained snow	0.18	[0.03, 0.33]	0.12	[-0.33, 0.57]	0.2	[0.04, 0.34]	-0.07	[-0.54, 0.40]	**-0.39**	**[-0.59, -0.17]**
Moist transformed snow	0.17	[0.01, 0.27]	0.26	[-0.18, 0.60]	0.15	[0, 0.27]	0.11	[-0.33, 0.48]	**-0.39**	**[-0.58, -0.19]**
Wet corn snow	0.00	[-0.13, 0.18]	0.26	[-0.18, 0.59]	-0.02	[-0.15, 0.16]	0.29	[-0.20, 0.65]	**-0.39**	**[-0.58, -0.20]**
Very wet corn snow	-0.14	[-0.23, 0.05]	-0.07	[-0.54, 0.49]	-0.13	[-0.21, 0.05]	0.06	[-0.46, 0.60]	**-0.40**	**[-0.60, -0.18]**
Q7 Average	0.26	[0.20, 0.34]	0.37	[0.10, 0.61]	0.25	[0.19, 0.33]	0.12	[-0.16, 0.37]	**-0.41**	**[-0.56, -0.26]**
Q8: For racing classic skis, which ski property or preparation factor has the greatest effect on glide in each snow condition?										
New snow	-0.11	[-0.35, 0.12]	-0.46	[-0.91, 0.39]	-0.07	[-0.33, 0.18]	-0.39	[-0.95, 0.50]	-0.25	[-0.47, -0.01]
Fine grained snow	0.25	[0.09, 0.38]	-0.06	[-0.68, 0.59]	0.25	[0.1, 0.39]	-0.31	[-0.94, 0.36]	-0.28	[-0.51, -0.04]
Transformed snow	0.30	[0.16, 0.41]	0.07	[-0.56, 0.65]	0.30	[0.17, 0.42]	-0.23	[-0.88, 0.37]	**-0.36**	**[-0.58, -0.12]**
Frozen wet snow	0.17	[-0.06, 0.35]	0.07	[-0.55, 0.65]	0.18	[-0.05, 0.37]	-0.11	[-0.75, 0.53]	**-0.34**	**[-0.55, -0.11]**
Moist fine-grained snow	0.06	[-0.13, 0.22]	-0.11	[-0.56, 0.52]	0.08	[-0.12, 0.23]	-0.19	[-0.66, 0.46]	**-0.35**	**[-0.58, -0.12]**
Moist transformed snow	0.27	[0.14, 0.37]	0.22	[-0.35, 0.61]	0.27	[0.15, 0.37]	-0.06	[-0.62, 0.36]	**-0.38**	**[-0.6, -0.14]**
Wet corn snow	0.30	[0.18, 0.41]	0.09	[-0.43, 0.57]	0.30	[0.17, 0.41]	-0.21	[-0.75, 0.29]	-0.26	[-0.48, -0.02]
Very wet corn snow	0.26	[0.14, 0.38]	0.19	[-0.32, 0.53]	0.27	[0.14, 0.39]	-0.08	[-0.62, 0.28]	-0.26	[-0.50, -0.01]
Q8 Average	0.19	[0.08, 0.28]	0.00	[-0.41, 0.47]	0.20	[0.09, 0.29]	-0.20	[-0.62, 0.29]	**-0.31**	**[-0.50, -0.11]**
Q9: You have the best possible skis and waxes. Now choose between 4 different stone grinds. Which one do you select to get the best glide for each snow type?										
New snow	0.73	[0.66, 0.78]	0.77	[0.67, 0.90]	0.72	[0.65, 0.78]	0.05	[-0.07, 0.20]	-0.22	[-0.39, -0.04]
Fine grained snow	0.64	[0.60, 0.70]	0.77	[0.67, 0.90]	0.63	[0.59, 0.69]	**0.14**	**[0.02, 0.28]**	**-0.37**	**[-0.54, -0.19]**
Transformed snow	0.73	[0.67, 0.78]	0.83	[0.70, 0.96]	0.72	[0.65, 0.78]	0.12	[-0.03, 0.26]	**-0.27**	**[-0.42, -0.10]**
Frozen wet snow	0.46	[0.37, 0.55]	0.67	[0.48, 0.85]	0.43	[0.34, 0.54]	**0.23**	**[0.02, 0.44]**	-0.17	[-0.34, 0.00]
Moist fine-grained snow	0.69	[0.62, 0.75]	0.80	[0.62, 0.96]	0.67	[0.6, 0.74]	0.13	[-0.06, 0.30]	-0.21	[-0.37, -0.04]
Moist transformed snow	0.76	[0.70, 0.82]	0.78	[0.48, 1.00]	0.75	[0.69, 0.81]	0.03	[-0.28, 0.26]	**-0.28**	**[-0.41, -0.13]**
Wet corn snow	0.80	[0.75, 0.85]	0.67	[0.54, 0.84]	0.82	[0.76, 0.87]	-0.15	[-0.28, 0.03]	**-0.39**	**[-0.57, -0.20]**
Very wet corn snow	0.94	[0.89, 0.97]	0.86	[0.61, 1.00]	0.94	[0.88, 0.98]	-0.08	[-0.34, 0.06]	**-0.35**	**[-0.50, -0.21]**
Q9 Average	0.72	[0.69, 0.75]	0.77	[0.70, 0.85]	0.71	[0.68, 0.74]	0.06	[-0.02, 0.14]	**-0.28**	**[-0.36, -0.19]**
Q10: You have the best possible skis and stone grinds. Now choose between 5 glide waxes of varying hardness. Which one do you select to get the best glide for each snow type?										
New snow	0.34	[0.24, 0.42]	0.60	[0.48, 0.79]	0.31	[0.20, 0.40]	**0.29**	**[0.15, 0.52]**	-0.15	[-0.32, 0.02]
Fine grained snow	0.46	[0.40, 0.53]	0.60	[0.38, 0.78]	0.45	[0.38, 0.53]	0.15	[-0.09, 0.34]	-0.24	[-0.43, -0.04]
Transformed snow	0.61	[0.54, 0.67]	0.68	[0.54, 0.86]	0.6	[0.53, 0.67]	0.07	[-0.07, 0.27]	-0.21	[-0.39, -0.02]
Frozen wet snow	0.66	[0.60, 0.72]	0.65	[0.53, 0.82]	0.66	[0.59, 0.73]	-0.01	[-0.15, 0.17]	**-0.30**	**[-0.49, -0.12]**
Moist fine-grained snow	0.54	[0.47, 0.62]	0.70	[0.50, 0.91]	0.53	[0.46, 0.61]	0.17	[-0.04, 0.39]	-0.05	[-0.21, 0.12]

Moist transformed snow	0.55	[0.47, 0.62]	0.55	[0.29, 0.79]	0.56	[0.48, 0.64]	0.00	[-0.27, 0.24]	-0.12	[-0.33, 0.08]
Wet corn snow	0.36	[0.24, 0.45]	0.22	[-0.16, 0.56]	0.37	[0.26, 0.47]	-0.15	[-0.55, 0.21]	-0.17	[-0.38, 0.04]
Very wet corn snow	0.47	[0.31, 0.60]	0.07	[-0.41, 0.53]	0.51	[0.35, 0.64]	-0.44	[-0.92, 0.05]	-0.12	[-0.33, 0.10]
Q10 Average	0.50	[0.44, 0.55]	0.51	[0.37, 0.67]	0.50	[0.44, 0.55]	0.01	[-0.14, 0.18]	-0.17	[-0.28, -0.05]

Supplementary Table 12. Agreement by snow type (ST1–ST8; see Table 1) for the full sample, experts, and non-experts, and the expert–non-expert difference. Agreement is quantified using van der Eijk's agreement coefficient (A) for ordered rating data [39]. Values are reported as the median (Med) with a 95% bootstrap confidence interval (CI; 10,000 resamples). Group difference is defined as $A_{\text{expert}} - A_{\text{non-expert}}$(positive values indicate higher agreement among experts). The *Average* is the unweighted mean that summarizes results across questions within each snow type. Boldfaced values indicate group-difference estimates with 95% CIs that exclude zero.

	Agreement: Full sample		Agreement: Experts		Agreement: Non-experts		Agreement Group difference	
	Med	95% CI	Med	95% CI	Med	95% CI	Med	95% CI
ST1: New snow	0.28	[0.22, 0.34]	0.29	[0.17, 0.50]	0.29	[0.22, 0.35]	0.00	[-0.13, 0.22]
ST2: Fine grained snow	0.49	[0.45, 0.53]	0.48	[0.37, 0.63]	0.48	[0.44, 0.52]	0.00	[-0.12, 0.16]
ST3: Transformed snow	0.51	[0.48, 0.54]	0.57	[0.49, 0.66]	0.51	[0.47, 0.54]	0.07	[-0.02, 0.16]
ST4: Frozen wet snow	0.58	[0.54, 0.62]	0.59	[0.49, 0.68]	0.58	[0.53, 0.62]	0.01	[-0.10, 0.12]
ST5: Moist fine-grained snow	0.27	[0.24, 0.33]	0.33	[0.23, 0.51]	0.27	[0.24, 0.34]	0.06	[-0.07, 0.23]
ST6: Moist transformed snow	0.45	[0.41, 0.49]	0.52	[0.46, 0.63]	0.44	[0.40, 0.49]	**0.08**	**[0.00, 0.20]**
ST7: Wet corn snow	0.42	[0.37, 0.47]	0.42	[0.32, 0.54]	0.41	[0.37, 0.47]	0.01	[-0.11, 0.13]
ST8: Very wet corn snow	0.47	[0.43, 0.53]	0.47	[0.34, 0.59]	0.49	[0.44, 0.54]	-0.02	[-0.16, 0.11]
Average	0.43	[0.39, 0.48]	0.46	[0.36, 0.59]	0.43	[0.39, 0.48]	0.03	[-0.09, 0.17]
